# Public transport across models and scales: A case study of the Munich network

**DOI:** 10.1093/pnasnexus/pgae489

**Published:** 2024-10-31

**Authors:** Jan Mölter, Joanna Ji, Benedikt Lienkamp, Qin Zhang, Ana T Moreno, Maximilian Schiffer, Rolf Moeckel, Christian Kuehn

**Affiliations:** Department of Mathematics, School of Computation, Information and Technology, Technical University of Munich, Boltzmannstraße 3, Garching bei München, 85748 Germany; Department of Mobility Systems Engineering, School of Engineering and Design, Technical University of Munich, Arcisstraße 21, Munich 80333, Germany; Department of Operations & Technology, School of Management, Technical University of Munich, Arcisstraße 21, Munich 80333, Germany; Department of Mobility Systems Engineering, School of Engineering and Design, Technical University of Munich, Arcisstraße 21, Munich 80333, Germany; Department of Mobility Systems Engineering, School of Engineering and Design, Technical University of Munich, Arcisstraße 21, Munich 80333, Germany; Department of Operations & Technology, School of Management, Technical University of Munich, Arcisstraße 21, Munich 80333, Germany; Munich Data Science Institute, Technical University of Munich, Walther-von-Dyck-Straße 10, Garching 85748, Germany; Department of Mobility Systems Engineering, School of Engineering and Design, Technical University of Munich, Arcisstraße 21, Munich 80333, Germany; Munich Data Science Institute, Technical University of Munich, Walther-von-Dyck-Straße 10, Garching 85748, Germany; Department of Mathematics, School of Computation, Information and Technology, Technical University of Munich, Boltzmannstraße 3, Garching bei München, 85748 Germany; Munich Data Science Institute, Technical University of Munich, Walther-von-Dyck-Straße 10, Garching 85748, Germany; Complexity Science Hub Vienna, Josefstädter Straße 39, Vienna 1080, Austria

**Keywords:** public transport, mathematical modeling, simulation, optimization

## Abstract

The use of public transport systems is a striking example of complex human behavior. Modeling, planning, and managing public transport is a major future challenge considering the drastically accelerated population growth in many urban areas. The desire to design sustainable cities that can cope with a dynamically increasing demand requires models for transport networks since we are not able to perform real-life experiments before constructing additional infrastructure. Yet, there is a fundamental challenge in the modeling process: we have to understand which basic principles apply to the design of transit networks. In this work, we are going to compare three scientific methods to understand human behavior in public transport modeling: agent-based models, centralized optimization-based models, and minimal physics-based models. As a case study, we focus on the transport network in Munich, Germany. We show that there are certain universal macroscopic emergent features of public transport that arise regardless of the model chosen. In particular, we can obtain with minimal basic assumptions a common and robust distribution for the individual passenger in-vehicle time as well as for several other distributions. Yet, there are other more microscopic features that differ between the individual and centralized organization and/or that cannot be reproduced by a minimal nonlocal random-walk type model. Finally, we cross-validate our results with observed public transport data. In summary, our results provide a key understanding of the basic assumptions that have to underlie transport modeling for human behavior in future sustainable cities.

**Significance Statement**

With the increasing human population, public transport systems will be an integral component of cities in the future. In this study, we introduce and compare three conceptually different models of human mobility in the context of public transport. These include a detailed agent-based simulation, a centralized optimization-based model, and a minimal statistical model. As the underlying public transport system, we consider the Munich bus network. It is comprised of almost one thousand stations and thus is representative of a public transport system in a major European city. Importantly, by comparing macroscopic observables, such as the time individuals spend on a bus during a trip, we show that certain universal emergent features arise regardless of the modeling approach.

## Introduction

How should we organize the public transport systems of the future? Should we just build some reasonable infrastructure and then leave its usage to agent-based self-organization? Or should we centrally optimize paths by specific and adaptive route suggestions provided upon the request of a travel demand? As for many other large scale complex systems (1), e.g. climate or ecosystems (2), it is difficult to answer these questions by experimenting on transport systems. We cannot just experimentally test different instances repeatedly on a large scale as it is prohibitively costly and time-consuming to (re-)design a full public transport network and may result in unintended negative effects. Therefore, developing reliable models and design principles a priori is of paramount importance. This raises the question of modeling scale and starting assumptions. Perhaps it suffices to assume a very simple, potentially statistically universal, model of human mobility. In this work, we will compare different models of human mobility in the context of public transport networks using the city of Munich, Germany as a case study, and cross-validate these models with real-world data. We compare three different modeling approaches ranging from an agent-based simulation, and a global-optimization exogenous-controlled setting, to an abstract statistical physics random-walk model.

These three modeling approaches effectively span the most common techniques and disciplines currently employed to model transport dynamics. Fundamentally, agent-based systems are micro-simulations (3) where the behavior of each individual is modeled separately. Agent-based models specifically allow for the interaction of agents (4), where choices made by one agent may affect the choices of other agents. The first agent-based models in transport were proposed by (5), where agents have an activity schedule that requires traveling to conduct activities at different locations. While some agent-based transport models use trips as the unit of analysis (e.g. MITO (6)), others use an activity-based paradigm (e.g. ActivitySim (7), OASIS (8), SimMobiliy (9), TASHA (10)). (11) provides a good overview of the state-of-the-art. In this work, we generate travel demand with MITO (6) and assign trips to a multimodal network with MATSim (12).

Contrary to agent-based models, multicommodity network flow (MCNF) problems abstract from an individual’s behavior and consider a system-centric perspective, i.e. central control over all activities in the network. Such an approach allows leveraging techniques from continuous and discrete optimization to compute the system optimum, i.e. a globally optimal solution under the assumption that all individuals contribute to the system’s optimal objective. MCNF problems have been studied in the field of operations research for decades, with a multitude of solution techniques that range from heuristic (see, e.g. (13)) to exact (see, e.g. (14)) algorithms. MCNF problems have recently often been used to analyze transportation systems from a mesoscopic perspective (see, e.g. (15)) and are flexible in terms of modeling additional characteristics and constraints, e.g. a time dimension (16) or intermodal transportation (17). Moreover, MCNF modeling approaches allow optimizing transport network design decisions with reasonable computational effort at the price of approximating individual behavior via the system optimum (18).

Finally, in the most abstract level, transport can be modeled using tools from nonlinear dynamics and statistical physics (19, 20). Random walks are among the simplest stochastic processes that have been used across a wide range of modeling scenarios and they are generally rather well understood (21–24). In the traditional setting, the dynamics are entirely local, meaning that the walker only perceives its immediate neighborhood. Yet, biased and nonlocal random walks have been increasingly investigated in the last few decades because of their relevance in mobility and transportation models and navigation (25–29). While there are studies that consider some specific non-Markovian random walks (30–33), in the context of modeling mobility, a shortcoming of the usual models is their memorylessness. In this work, we therefore employ a minimal Inertial Random Walk (IRW) model on a network to account for the tendency of target-oriented movement in human mobility in general and public transport in particular.

The paper is structured as follows. First, we provide more details on our modeling approaches to human behavior in public transport, starting from agent-based endogenous behavior, discussing global optimization as exogenous decision support, and finally defining suitable minimal statistical physics models. Then, we report our main results: These include the emerging universal structures of macroscale probability distribution observables but also subtle differences between model results. In particular, we discuss the unimodal common shape for in-vehicle times and trip distances, as well as the high correlation between distributions of passengers at stations/stops. Then, we compare these results to public transport data from the Munich metropolitan area. Finally, we interpret our results for the future design of public transport models and their practical implications for building sustainable cities.

## Results

Before presenting our main computational and data-based results, a key aspect of our work is to provide a cross-cutting comparison for different models across scales. Therefore, we start with a description of our methodology.

As a first component, our work uses agent-based travel demand generated by the model MITO (6). A synthetic population was generated for the Munich region (34) that provides a statistically equivalent representation of each person of the actual population. MITO uses discrete choice and hazard-duration models to simulate a number of trips, destination choice, mode choice, and departure/arrival time choice. This serves as the input travel demand for the first two approaches.

In the first approach, this travel demand is assigned to a multimodal network using MATSim (12). MATSim is a multiagent transport simulation system that can be used as a Dynamic Traffic Assignment (DTA) model that simulates individual vehicles on the road network (35). Every agent optimizes their daily activity schedule while competing for space-time slots on the transportation networks with other agents. MATSim is used to select routes on multimodal transport networks, respecting travel demand input by MITO.

Yet, a drawback of purely agent-based models is that they are endogenously driven, so that agents aim to self-organize and target first their own demands. Against this background, one may aim to study transportation networks through the lens of a central decision-maker that controls all flows in the system. Such a perspective is particularly valuable in order to obtain an upper bound on the improvement potential that can be reached by global coordination measures or in order to optimize network design decisions under the assumption that once network capacities are established, individual behavior will step-wise converge to the system optimal behavior. To compute such system optimal solutions, in this approach, we leverage MCNF problems, which receive origin–destination pairs of all individuals that aim to travel in a network as input and compute the optimal flow for each individual through the network according to some global cost metric that is induced by all resulting flows. Such MCNF problems can be subject to additional constraints that influence the difficulty of the problem. For example, one can model MCNF problems with fractional or integer flows as well as with and without capacity constraints on a network’s connections. While the uncapacitated problem variant remains computationally solvable in polynomial time, the integer flow variant is computationally hard and requires advanced algorithmic solution techniques. Beyond these basic differences, MCNF problems can be solved as time-variant problems, e.g. by using a temporal network expansion, or as time-invariant problems on a flat network.

Agent-based modeling simulation, as well as global optimization, can often be costly in terms of model building and simulation time. Furthermore, it is difficult to discern in these approaches which role certain details of the model and the parameter choices play due to the high complexity. On the contrary, nonlinear dynamics and statistical physics provide robust, generic, and simple models for movement, which one can apply to transport problems. In the third approach, as a minimal model for human mobility in a public transport network, we constructed a random-walk-type model. However, human mobility tends to be characterized by non-Markovian, i.e. not memoryless, dynamics, so a standard random walk does not suffice. For instance, where in a standard random walk a walker may jump back and forth between adjacent sites, this is very unlikely to be observed in human mobility patterns. Instead, a human walker will continue to move in the same direction while traversing several stations and only change direction at major transportation hubs. Another aspect of human mobility patterns is that excursion lengths follow certain empirical distributions that might depend on the overall layout of the transport network. Both of these aspects are generally incompatible with Markovian dynamics and require the walker to have some form of memory of where it came from and how long it was already walking. To capture these behaviors, in the random-walk-type model we propose, we combine an inertial random walk on a public transport network with a resetting mechanism that depends on the number of steps previously taken. This yields a process that is highly non-Markovian. However, instead of directly constructing this process, here we consider a higher-order, layered network constructed from the underlying public transport network so that a standard random walk on this expanded network appears to have the non-Markovian characteristics when projected onto the public transport network. In that way, we retain the analytical and computational simplicity of a random walk, albeit on a much larger and more complicated network, and at the same time, can produce dynamics that are strictly non-Markovian.

For more technical details on the models, MATSim, MCNF, and IRW, refer to the Methods.

**Fig. 1.**
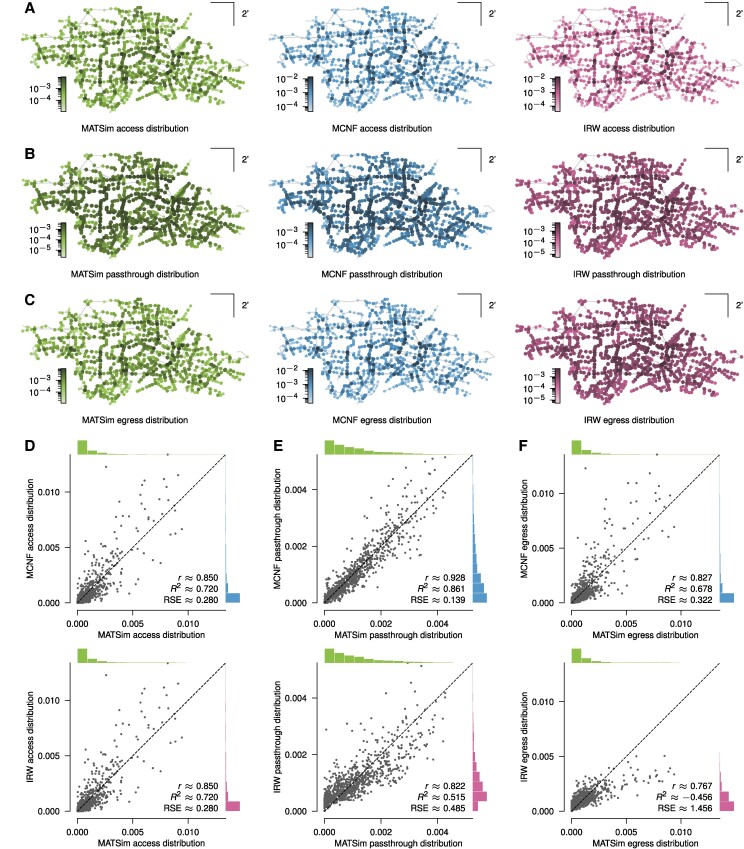
Station distributions. A, B, C) Access, passthrough, and egress distributions, respectively, for the MATSim, MCNF, and IRW models, showing the probability with which any station is the startpoint, an intermediate point, and the endpoint of a trip. D, E, F) Comparison of the access, passthrough, and egress distributions, respectively, for the individual stations of the MCNF and IRW models with the MATSim model as a reference. — r: The Pearson correlation coefficient between the sample points; R^2^, RSE: The coefficient of determination and relative square error, respectively, for a regression of the sample points on the 1:1 line.

Proceeding to our computational results, we want to compare the three modeling approaches. We begin by looking at the emerging access, passthrough, and egress distributions for each of the three modeling approaches (Fig. 1A–C). The access and egress distribution measure the frequency (or probability) with which the different stations are the access and egress points to the bus network, i.e. the start- or endpoint of a bus trip, respectively. The passthrough distribution measures the frequency with which any given station was traversed. When comparing the results, we found that the access distributions of the MATSim model were slightly flatter than the MCNF and IRW models, both of which tended to produce more hubs that manifest in longer tails of the marginals (Fig. 1D). Note that the MCNF and IRW had the same access distributions (see Methods). The passthrough distributions were very similar for the MATSim and MCNF models, whereas in the IRW model, the proportion of stations with lowest passthrough frequency is considerably lower (Fig. 1E). Finally, the egress distributions of the MATSim and MCNF models were again quite similar, except for possibly a few additional hubs in the latter. Conversely, the IRW model egress distribution was significantly different in its flatter nature (Fig. 1F). The overall difference between the passthrough and egress distributions of the MATSim and MCNF models versus the IRW model likely stems from the latter spreading the trips more evenly across the whole network, leading to the flatter distributions. In contrast, the MATSim and MCNF models account for nonuniform travel demand across the network area, leading to distributions that are less flat.

**Fig. 2.**
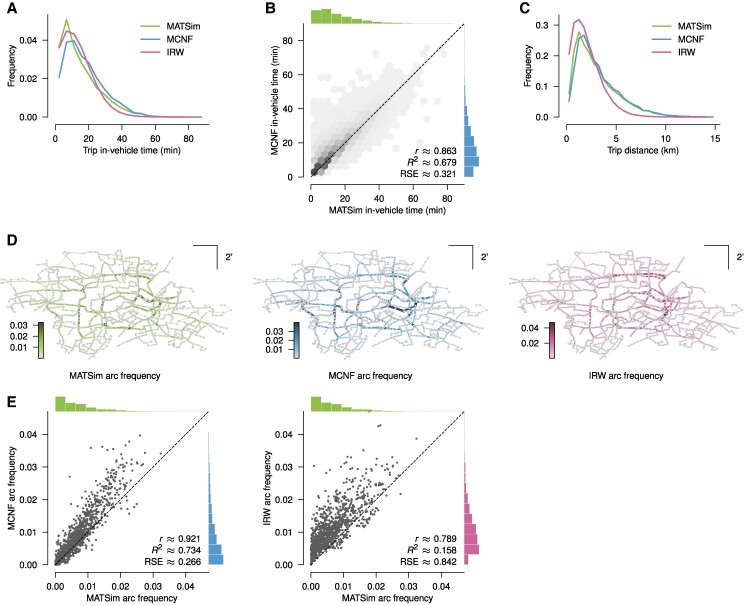
Trip properties. A) Distribution of in-vehicle times. The overall shape is relatively similar for all three models. B) Direct comparison of in-vehicle times per trip of the MATSim and MCNF model showing that the latter produced slightly longer trips. C) Distribution of trip distances, i.e. the straight distances between trip endpoints. Due to having the same travel demand, the distributions of the MATSim and MCNF models were essentially the same, whereas the IRW model produced trips that covered less distance. D) Arc frequency for the MATSim, MCNF, and IRW models, showing the frequency with which different arc were used along trips. In each case, clear favored routes emerge. E) Comparison of arc frequencies for the individual arcs of the MCNF and IRW models with the MATSim model as a reference. — r: The Pearson correlation coefficient between the sample points; R^2^, RSE: The coefficient of determination and relative square error, respectively, for a regression of the sample points on the 1:1 line.

Next, we looked more closely at the individual trips that each of the models produced. Importantly, the distribution of in-vehicle times, i.e. the cumulative time a passenger spent inside a bus, was overall relatively similar for all three models (Fig. 2A). However, serving the same travel demand also allowed a direct comparison of in-vehicle times for each individual trip between the MATSim and MCNF models. This revealed that the latter produced slightly longer trips (Fig. 2B). In terms of trip distances, i.e. the straight line distance between the trip’s endpoints, the distributions for MATSim and MCNF models were almost the same (due to the same travel demand), whereas the IRW model produced trips that covered comparatively less distance (Fig. 2C). This is essentially because the IRW model implements local dynamics. Lastly, considering the frequency with which different arcs of the network were used along trips generated with the three models revealed clear routes that supported a majority of trips. While there was some overlap where two models gave rise to the same routes, distinct differences were evident between any two models (Fig. 2D). In direct comparison, the arc frequencies of the MATSim and the MCNF model were relatively similar, with MCNF frequencies slightly higher than the MATSim frequencies overall, while the IRW frequencies were considerably higher for almost every arc (Fig. 2E).

**Fig. 3.**
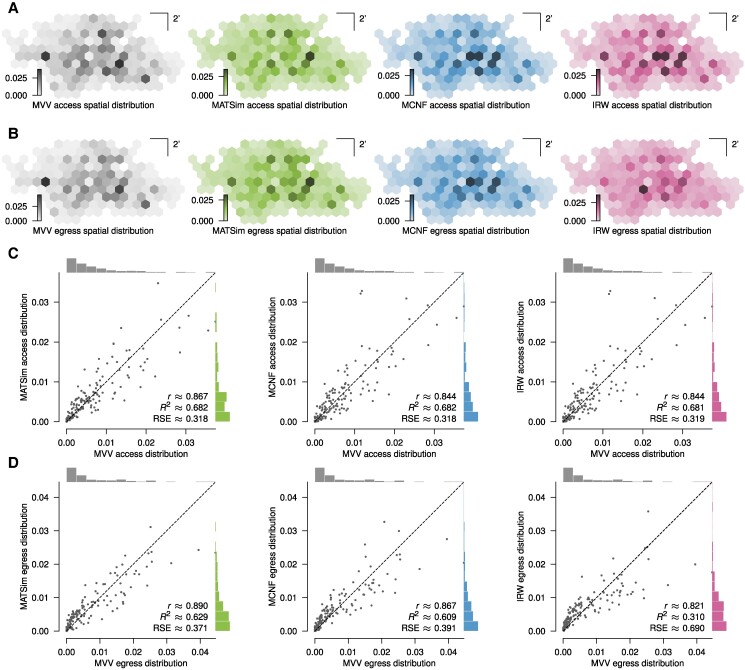
Model validation with data. A, B) Access and egress spatial distributions, respectively, for the MVV data and the MATSim, MCNF, and IRW models, showing the probability of a neighborhood as the startpoint or the endpoint of a trip. C, D) Comparison of the access and egress spatial distributions, respectively, for the individual neighborhoods of the MVV data and the MATSim, MCNF, and IRW models with the MVV data as a reference. — r: The Pearson correlation coefficient between the sample points; R^2^, RSE: The coefficient of determination and relative square error, respectively, for a regression of the sample points on the 1:1 line.

Finally, we compared the models’ output with actual data provided by the Munich Transport and Tariff Association (MVV), the Munich transport authority. These data were collected from 2019 October 7 to 2019 December 20, and encompasses the average number of passengers accessing and egressing at each bus stop in the network from Monday to Friday, from the start to end of service. We compared this with the MATSim, MCNF, and IRW models. However, since it was not possible to unambiguously map these distributions onto the bus network we used before, we instead discretized the area of the network and considered trips between neighborhood cells rather than individual bus stops and the corresponding distributions. Overall, the distributions turned out very similar, presenting the same hotspots (Fig. 3A,B). This is also reflected in a pointwise comparison (Fig. 3C,D).

In summary, comparing the three modeling approaches on a Munich public transport network and cross-validating the results against observed transport data, we found as a first key result that several features are surprisingly universal. In particular, several macro-level probability distributions are in excellent agreement between all of them. This includes access (passengers boarding transit at a given station), passthrough (passengers remaining on the transit vehicle at a given station), and egress (passengers alighting at a given station) distributions of the stations/stops. Furthermore, the in-vehicle time as well as the trip distance exhibit universal unimodal distributions. In particular, this proves that the outcome for key macroscopic observables of public transport are not dictated by the modeling scales/approaches, but are essentially shaped by the underlying network structure and travel demands. These findings align with the Munich public transport dataset. Yet, once we leave the macroscale, the three models show certain microscopic differences, e.g. in slight preferences for certain routes or variations in in-vehicle time for particular trips.

## Discussion

Modeling approaches to build new or improve existing public transportation infrastructure are clearly of paramount importance to improve city infrastructure. Yet, one might have initially conjectured that the wide variety of approaches, such as agent-based models, global optimization, and conceptual nonlinear dynamics approaches, could significantly influence the outcomes relevant for decision-makers. Our study provides a clear indication that this may not be the case and one obtains very comparable results on a macroscopic level. This suggests that the assumptions and external constraints to our models should be viewed as the key data. In particular, we observed in our case study for the Munich network that there is a basic law of “supply” (the network topology of the routes) and “demand” (consisting of the travel demands of individuals). Once supply and demand are fixed, the practical management aspect of routing individual trips should be viewed as fine-tuning the system, highlighting specifically also the importance of the network topology as discussed in (36). Indeed, in this context, all three modeling approaches are likely to succeed, when we aim to fine-tune an existing supply–demand system of public transportation.

Therefore, our results suggest that accurate and reliable a priori estimates for travel demand within a city are crucial for designing or re-designing a public transportation network. Once the network topology is fixed by certain roads or rail tracks, and the city areas are fixed into commercial, residential, recreational, etc., then it will be very difficult to adjust a public transportation network as adding additional pathways into highly populated areas tends to be extremely costly. Yet, the positive conclusion from our study is that utilizing all three modeling approaches can lead to successful results if utilized and implemented correctly, i.e. once the input data of supply and demand are accurate, it will be possible to use models and simulations to find a robust network design.

To finally answer the question of which of the three modeling approaches should be used in practice, one needs to specify the problem to be studied and the (computing) resources available. If one is interested in microscopic details of traffic flows, it is worthwhile to consider agent-based models. In contrast, if one rather takes a macroscopic view and, for instance, only needs an aggregated cost estimate based on the global quantities, the coarser models will provide as good of an estimate as an agent-based model but come with less computational overhead.

More specifically, our results show that in cases where the decision maker is only interested in aggregated characteristics, it might well likely be sufficient to rely on our MCNF or random walk modeling approach, which can be beneficial when modeling transportation networks in a paramount modeling or optimization task. For example, one might rely on a random walk model that can be coupled with standard epidemic dynamics on (social) networks when analyzing the impact of public transport on the spreading of an epidemic (37). Similarly, one may rely on an MCNF model to approximate traffic flow dynamics when accounting for them in a game-theoretic context (17) or when making network design decisions. By doing so, one can utilize advanced optimization techniques for the paramount planning task. Still, one can afterward use a microscopic model to understand the dynamics of the resulting system in detail for a posteriori analyses and, if necessary, adaptions.

## Methods

### Construction of the public transport network

Networks for local public transport buses were acquired from the Germany-wide GTFS feeds aggregator (38). Downloadable data included stop locations, lines (in the sequence of stops), and journeys (individual services of each line on a selected day). The timetable information encompasses a comprehensive all-day schedule. The data were subsequently converted using the pt2matsim framework (39).

For the MCNF and IRW models, we derive the flat bus network consisting of 986 nodes and 2,228 arcs based on these converted data. To do so, we filter for all bus stops with coordinates in the polygon of the city of Munich, map the coordinates of the stops to the name of the nearest stop in the GTFS data, and merge all stops with identical names such that the merged stop nodes’ coordinates represent the center of the, respectively, merged stops. We derive the polygon of the city of Munich through OSMnx (40). If any bus route exists between a stop pair, we add an arc between those two stops. This procedure can be reproduced with instance_generator.py in (41).

### Models

In the following, we describe the technical details of the three models we considered.

#### Travel demand generation

The travel demand used in the MATSim and MCNF models was generated using the agent-based travel demand model MITO (Microscopic Transportation Orchestrator) (6). The open-source model (https://github.com/msmobility/mito) works as a microsimulation and generates travel demand individually for every person in every household of a study area’s synthetic population. Here, the synthetic population was generated for the Munich metropolitan area as documented in (34). The model was estimated based on the German national household travel survey (42).

MITO consists of four modules that create travel demand.

1. First, we select the number of trips for a given person using a hurdle model. First, the model estimates the probability of performing no trips (binary logistic regression model). Second, if the person performs any trip, a truncated negative binomial regression is used to obtain the count of trips.
2. For destination choice, work, and school locations are defined in the synthetic population. All other destinations are selected with a logit-based destination choice model that reflects travel distance and attractiveness of destinations. The utility between each possible origin and destination relation for every trip purpose is defined as follows:
  $$e^{U_{i\;|\;j}}=e^{\beta^{*}\cdot {\text{imp}}_{i\;|\;j}+\ln\;(\text{attraction})}=e^{\beta^{*}\cdot {\text{imp}}_{i\;|\;j}}\cdot \text{attraction},$$
  where $e^{U_{i\;|\;j}}$ is the exponential utility of choosing destination *j* from origin *i* for trip purpose *p*, and ${\text{imp}}_{i\;|\;j\;|\;p}$ is the impedance for this trip. The attraction variable is estimated during the trip generation phase and represents the quantity of opportunities available in the destination zone corresponding to the specific trip purpose. The magnitude of the impedance is determined by the purpose-specific parameter *β*. Impedance is computed as follows:
  $${\text{imp}}_{i\;|\;j}=e^{{\mathrm{td}}_{i\;|\;j}}\cdot c_{p},$$
  with ${\text{td}}_{i\;|\;j}$ being the travel distance between origin *i* and destination *j*, and $c_{p}$ being a calibrated parameter for each trip purpose *p*. Travel distances are used for the impedance. The parameters *β* and $c_{p}$ are calibrated to match the distribution and the average reported trip distances for each purpose in the household travel survey. The parameters *β* and $c_{p}$ are calibrated parameters to align with the distribution and average trip distances in the German household travel survey.
3. Mode choice is selected via a nested logit discrete choice model. The model selects the travel mode for each trip purpose based on supply characteristics, trip characteristics, and traveler attributes. The model chooses among modes Auto driver, Auto passenger, Bicycle, Bus, Train, Tram/Metro, and Walk, with Auto driver and Auto passenger grouped under Auto nest and Bus, Train, Tram/Metro grouped under Transit. The probability of choosing an alternative is calculated by the following equation:
  $$\text{Pr}(i)=\frac{e^{V_{i}}}{\sum_{\;j=1}^{J}e^{V_{j}}}$$
  where Pr(i) is the probability of choosing alternative *i*, and $V_{j}$ is the observable component of utility of alternative *j*. For nested modes, the probability is based on the conditional probability of choosing the nested mode multiplied with the probability of choosing the nest. The observable portion of the utility is calculated as follows:
  $$V_{i,t}=V(S_{t})+V(X_{i})+V(S_{t},X_{i}),$$
  where $V_{i,t}$ is the observable portion of utility of alternative *i* for individual *t*, $V(S_{t})$ is the part of utility associated with characteristics of individual *t*, $V(X_{i})$ is the utility from the attributes of alternative *i*, and $V(S_{t},X_{i})$ is the utility from interactions between the attributes of alternative *i* and the characteristics of individual *t*.
4. The preferred arrival time is provided by the synthetic population for work and school trips. For nonmandatory trips, the preferred departure time is chosen probabilistically based on observed arrival time distributions.

The above modules were all calibrated to match the observed data. The result of MITO is a list of trips that describe the travel demand of every agent in a given study area. As MITO works microscopically, attributes of individual travelers, such as auto ownership, access to car sharing, disability, income, smartphone availability, etc., all may influence travel decisions.

For this work, we generated the travel demand by allowing only buses as a mode of transport for a weekday between 6 AM and 2 PM but restricted the analysis to the time window from 7 AM to 1 PM For the comparison with data provided by the Munich Transport and Tariff Association (MVV), the Munich transport authority, the travel demand was generated again, allowing all possible modes of transport, which includes bus, tram, and subway (although we only compared trips that utilized buses exclusively) again for a weekday between 6 AM and 2 PM with an analysis time window from 7 AM to 1 PM To keep the simulation times manageable, the population was scaled down to 25% and 5%, respectively (43).

#### I: MATSim

Travel times from point to point were calculated using the SBB router within MATSim (44) based on GTFS data. For access and egress, MATSim considers walking on a road network acquired from OpenStreetMap (45). These road networks include freeways, trunk roads, and primary, secondary, and tertiary roads. MATSim provides very detailed simulation of public transport. Transit vehicles run along the predefined transit line routes and pick up and drop off passengers at stop locations. The transit vehicles operate based on the predefined time schedule, while road traffic conditions can also delay the arrival time of transit vehicles (e.g. buses) at stops. In the meanwhile, the capacities of transit vehicles are monitored during the simulation, which will affect the route decision of passengers.

The simulation generates transit-related events whenever a transit vehicle arrives or departs at a stop when passengers enter or leave a vehicle, but also when a passenger cannot board a vehicle because its capacity limit is already reached. Passengers in MATSim interact with transit vehicles. They must decide their routes based on transit services. For selecting the best transit route, several steps are carried out for each passenger. First, a set of possible access and egress transit stops are generated by searching all stops around the trip origin and destination within a certain distance threshold. Then more realistic transit routes are created based on the combinations of start and end stops. After that, a public transport router calculates the best route to the desired destination with minimal cost, given a departure time. Costs are typically defined as travel time and a small penalty for the number of transfers. Travel time of public transport trips consists of access time, egress time, waiting time and in-vehicle time. Finally, the cost of the best transit route is compared to the cost of direct walking from origin to destination, resulting in the final route choice of the passenger. As a result, the transit passengers in MATSim could eventually switch from taking the bus to direct walking if the cost of taking the bus is relatively high.

For this work, as key simulation parameters that were set before assigning public transport trips in MATSim, we had that access and egress stop search radius was 1 km in Euclidean distance from the coordination of trip start and end locations, the average walk speed used for calculating travel cost was 4 km/h, the penalty of number of transfers was 300 s, which means one transfer is equivalent to 5 min travel time, and that passengers had a fixed departure time. The capacities for different public transit modes are set to 60, 215, and 940, respectively, for bus, tram, and subway, according to the public transportation provider MVG (46).

#### II: Multicommodity Network Flow (MCNF)

In this model, we capture network-specific constraints, i.e. routes, travel times, and vehicle capacities, in a multilayered time-expanded graph and solve the problem of routing all passengers through the public transportation system, minimizing total travel time, via a minimum-cost multicommodity flow problem. To do so, we utilize the price-and-branch (P&B) approach proposed in (18), in which we decompose our initial problem into multiple shortest-path problems. We enhance our P&B approach with a pricing filter to reduce the number of solved pricing problems and an admissible distance approximation to utilize the $A^{*}$ algorithm for solving the pricing problems.

Our multilayered time-expanded digraph, representing the public transportation system, consists of one route layer for each route in the public transportation network, which allows passengers to use a vehicle that operates a route. Furthermore, waiting layers at each stop in the public transportation system enable passengers to wait at a stop for a new vehicle to arrive. Transit arcs between route and waiting layers allow passengers to enter or leave a vehicle at a stop. Finally, walking arcs between distinct waiting layers enable passengers to walk between stops within a certain distance. To formulate our problem of minimizing the total travel time as an optimization problem, we add access and egress arcs for each passenger connecting their origin and destination with stop layers of the public transportation system. For an in-depth explanation of the construction of the multilayered time-expanded digraph, we refer to Section 3.1 in (18).

We can now formulate our optimization problem as a standard MCNF problem. Accordingly, let G=(V,A) be the digraph representing our public transportation system with vertices *V* and arcs *A*. Let *P* be the set of passengers, where each p∈P is associated with a request tuple $\zeta_{p}=(o_{p},d_{p},\delta_{p})$ comprising an origin coordinate $o_{p}$, a destination coordinate $d_{p}$ and a departure time $\delta_{p}$, which is the timestep in which a passenger wants to begin its trip. Furthermore, let $\kappa_{ij}$ and $\tau_{ij}$ be the capacity, e.g. the capacity of a vehicle operating a route, and travel time of an arc (i,j)∈A respectively, $d_{i}^{p}$ be the vertex demand of vertex i∈V and passenger p∈P, and $x_{ij}\in \{0,1\}$ be the decision variable indicating whether passenger p∈P uses arc (i,j)∈A  $\left(x_{ij}=1\right)$ or not $\left(x_{ij}=0\right)$. The vertex demand of passenger p∈P in vertex i∈V is defined as


$$d_{i}^{p}=\left\{ \begin{array}{ll} 1 & \text{if}\;i=o_{p},\\ -1 & \text{if}\;i=d_{p},\\ 0 & \text{otherwise}. \end{array}\right.$$

Accordingly, we formulate our optimization problem as


(1*a*) $$\min_{x}\;\sum_{\;p\in P}\sum_{(i,j)\in A}\tau_{ij}\;x_{ij}^{p}\;\;\;\;\;\;\;\;$$

(1*b*) $$\;\;\;\text{s.t.}\;\sum_{\;j\in N^{+}(i)}x_{ij}^{p}-\sum_{\;j\in N^{-}(i)}x_{\;ji}^{p}=d_{i}^{p},\;\;\forall i\in V,p\in P\;\;\;\;\;\;\;\;\;\;\;\;$$

(1*c*) $$\sum_{\;p\in P}x_{ij}^{p}\leq \kappa_{ij},\;\;\;\;\;\;\forall (i,j)\in A$$

(1*d*) $$\;\;x_{ij}^{p}\in \{0,1\},\;\;\;\;\;\;\forall (i,j)\in A,p\in P.$$

Here, the objective function minimizes the sum over the travel time of all passengers, while Constraints (1b) ensure flow conservation with $N^{+}(i)$/$N^{-}(i)$ being the outgoing/ingoing neighborhood of vertex i∈V. Note that an ingoing neighbor of *i* is a vertex j∈V such that (j,i)∈A; an outgoing neighbor of *i* is a vertex *j* such that (i,j)∈A. Constraints (1c) enforce the capacity constraints of all arcs, and Constraints (1d) ensure integer passenger flows.

For large sets of passengers, solving Problem (1) becomes infeasible. Accordingly, we reformulate Problem (1) as a set-covering problem to solve it via a P&B approach. Here, we utilize column generation (CG) to solve the continuous relaxation of our MCNF problem and a commercial branch-and-bound solver to determine integer solutions. We formulate our set-covering formulation of the MCNF problem as


(2*a*) $$\min_{\lambda }\;\sum_{\;p\in P}\sum_{l\in L_{p}}\lambda_{l}^{\;p}\sum_{(i,j)\in A}c_{ij}(y_{l}^{p}{)}_{ij}$$

(2*b*) $$\;\;\;\;\;\text{s.t.}\;\sum_{\;p\in P}\sum_{l\in L_{p}}(y_{l}^{p}{)}_{ij}\lambda_{l}^{\;p}\leq \kappa_{ij}\;\forall (i,j)\in A$$

(2*c*) $$\;\;\;\;\;\;\;\;\;\;\;\;\;\;\;\;\;\;\;\;\;\;\sum_{l\in L_{p}}\lambda_{l}^{p}=1\;\;\;\;\;\;\;\;\;\;\;\;\;\;\;\;\;\;\;\;\forall p\in P$$

(2*d*) $$\;\;\;\;\;\;\lambda_{l}^{\;p}\in \{0,1\}\;\forall p\in P,l\in L_{p}.$$

Here, $L_{p}$ denotes the set of different paths that exist for a passenger p∈P. Parameter $(y_{l}^{p}{)}_{ij}$ shows whether path *l* of passenger *p* utilizes arc (i,j)∈A ($(y_{l}^{p}{)}_{ij}=1$) or not ($(y_{l}^{p}{)}_{ij}=0$) and we use decision variable $\lambda_{l}^{p}$ to select exactly one path $l\in L_{p}$ for each passenger *p*. As there may exist an exponential number of paths for each passenger, we relax Constraints (2d) to a nonnegativity constraint, introduce dummy path variables, and utilize CG to solve the resulting continuous relaxation of Problem (2). To do so, we iteratively solve the following pricing problem for each passenger p∈P.


(3*a*) $$\;\;\;\;\;\;\;\;\;\;\;\;\;\;\;\;\;\;\;\;\;\;\;\;\;\;\;\;\;\;\;\;\;\;\;\;\;\;\;\;\;\;\;\;\;\;\;\;\;\;\;\;\min_{y}\;\sum_{(i,j)\in A}(c_{ij}-w_{ij}^{*})y_{ij}^{p}-\alpha_{p}^{*}$$

(3*b*) $$\;\text{s.t.}\;\sum_{\;p\in P}y_{ij}^{p}\leq \kappa_{ij},\;\;\;\;\;\;\;\forall (i,j)\in A$$

(3*c*) $$\;\;\sum_{\;j\in N^{+}(i)}y_{ij}^{p}-\sum_{\;j\in N^{-}(i)}y_{\;ji}^{p}=d_{i}^{p},\;\forall i\in V,p\in P$$

(3*d*) $$\;\;\;\;y_{ij}^{p}\in \{0,1\},\;\;\;\;\;\;\;\forall (i,j)\in A,p\in P.$$

Here, $w_{ij}^{*}$ is the dual variable for the capacity constraints (2b) of arc (i,j)∈A and $\alpha_{p}^{*}$ the dual variable for the convexity constraint (2c) for passenger p∈P. Decision variables $y_{ij}^{p}$ determine whether the path we find for passenger *p* uses arc (i,j) ($y_{ij}^{p}=1$) or not ($y_{ij}^{p}=0$). Furthermore, Constraints (3c) ensure flow conservation for all vertices.

Due to our problem structure, we can solve Problem (3) as a shortest path problem. To do so, we use an admissible distance approximation that reduces the time-expanded multilayered digraph *G* to a significantly smaller static digraph $G^{\mathrm{static}}$, which allows us to utilize the $A^{*}$ algorithm. Furthermore, we utilize a pricing filter that significantly reduces the number of pricing problems we have to solve in each iteration of the CG. Finally, we solve the Restricted Master Problem of the last iteration of our CG with integer decision variables $\lambda_{l}^{\;p}$ to obtain integer solutions. We refer to Section 3.2 in (18) for an extensive overview of the entire P&B framework.

For this work, as key parameters, we used vehicle capacities based on information from the public transportation provider MVG (46), with capacity 60 for buses, 215 for trams, and 940 for subways. Furthermore, passengers can only enter and leave the public transportation system at stops within 1 km of their origin or destination, respectively. Here, passengers can only enter the public transportation system at stops where a vehicle leaves within 30 min of arriving at the stop. Lastly, we only add walking arcs between two stops with a maximum distance of 200 m.

#### III: Inertial Random Walk (IRW)

In this model, we combine an IRW on a public transport network with a resetting mechanism that depends on the number of steps previously taken. This naturally results in a process that is non-Markovian. To realize it, we construct a standard random walk on some higher-order network together with a projection onto the public transport network so that under the projection, the random walk on the higher-order network resembles the non-Markovian process.

We assume that the inertial movement can be described by a one-step memory transition kernel and that we are given some empirical distribution of the excursion lengths of a population of walkers as well as of the entry stations.

**Fig. 4.**
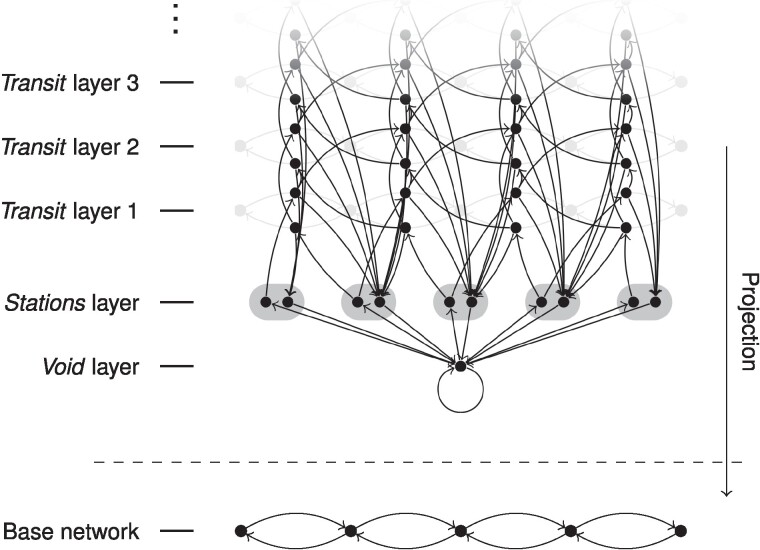
Construction of the IRW Markovian transition network. The Markov transition network associated with a base network is comprised of a void layer, a stations layer, and an infinite number of transit layers. The transitions go from the void node to the incoming stations’ nodes in the stations layer and from there into the stack of transit layers, which is an unfolding of the adjoint of the base network, with transitions between nodes in consecutive layers corresponding to adjacent links in the base network and transitions to the outgoing stations’ nodes in the stations layer and then to the void node. A node in the Markov transition network can be identified with a node in the base network via projection.

Then, given a directed, weighted transport network G=(X,A) with (weighted) adjacency matrix *A* and together with a one-step memory transition kernel *κ*, excursion length distribution *σ*, and access distribution *α*, the Markovian transition network is comprised of a *void* layer, a *stations* layer, and then stack of an, in general, infinite number of transit layers. The void layer consists only of a single node, *Ω*, which will be the origin and end of every excursion, the stations layer of two nodes for every node in the underlying transport network, $x_{\mathrm{in}},x_{\mathrm{out}}\;:\;x\in X$, to track incoming and outgoing walkers, and, finally, each transit layer of a node for every link in the underlying transport network, $(x,x^{\prime }{)}^{\left(l\right)}\;:\;(x,x^{\prime })\in A$ for layer *l* (Fig. 4).

These nodes will be connected via weighted, directed links within the same layer as well as between different layers, with the idea being that from the void node, there are transitions to the void node itself and to the incoming stations’ nodes in the stations layer according to the access distribution. From there, there are transitions into the stack of transit layers with transitions between nodes in consecutive layers corresponding to adjacent links in the base network. From every node in the stack of transit layers, there are in addition transitions back into the outgoing stations’ nodes in the stations layer and from there back to the void node.

If one ignores the fact that the transitions in the stack of transit layers are between consecutive layers, one has the transitions of a random walk on the adjoint of the base network. This provides the space to encode processes with one-step memory (47). Having this layered structure on top, allows one to track the number of steps already taken and thus arbitrary distributions of that through the transitions back into the stations layer.

Altogether, we have

- links $\Omega \to x_{\mathrm{in}}$ with weights α(x) for every x∈X,
- links $x_{\mathrm{in}}\to x_{\mathrm{out}}$ with weights $\left\{ \begin{array}{ll} 0 & \mathrm{if}\;{\text{deg}}_{\mathrm{out}}x>0,\\ 1 & \text{otherwise}. \end{array}\right.$ for every x∈X,
- links $x_{\mathrm{in}}\to (x,x^{\prime }{)}^{\left(1\right)}$ with weights $\frac{A_{xx^{\prime }}}{\sum_{\xi }A_{x\xi }}$ for every x∈X,
- links $(x,x^{\prime }{)}^{\left(l\right)}\to (x^{\prime },x^{\prime \prime }{)}^{\left(l+1\right)}$ with weights $\left(1-s_{l})\kappa (x^{\prime \prime }|x^{\prime },x\right)$ for every $\left(x,x^{\prime }\right)$, $(x^{\prime },x^{\prime \prime })\in A$,
- links $(x,x^{\prime }{)}^{\left(l\right)}\to x_{\mathrm{out}}^{\prime }$ with weights $\left\{ \begin{array}{ll} s_{l} & \mathrm{if}\;{\text{deg}}_{\mathrm{out}}x^{\prime }>0,\\ 1 & \text{otherwise}. \end{array}\right.$ for every $(x,x^{\prime })\in A$ and $x^{\prime }\in X$, and
- links $x_{\mathrm{out}}\to \Omega$ with weights 1 for every x∈X,

with $s_{l}=\left\{ \begin{array}{ll} \frac{\sigma_{l}}{\sum_{\lambda =l}^{\infty }\sigma_{\lambda }} & \mathrm{if}\;\sum_{\lambda =l}^{\infty }\sigma_{\lambda }>0,\\ 1 & \text{otherwise}. \end{array}\right.$ and where we assume that α(x)=0 if ${\text{deg}}_{\mathrm{out}}x=0$.

By construction, this network can be interpreted as a Markov transition network. In particular, this means that for every node in the network, the sum of the weights of the outgoing links is 1.

For the dynamics of the IRW, we concretely assume that the transition kernel is given as


(4) $$\begin{array}{l} \kappa_{\mathrm{inertial}}(x^{\prime \prime }|x^{\prime },x)=\left\{ \begin{array}{ll} \begin{array}{ll} (1-q)\delta_{x,x^{\prime \prime }}+q\frac{A_{x^{\prime }x^{\prime \prime }}}{\sum_{\xi }A_{x^{\prime }\xi }-A_{x^{\prime }x}}(1-\delta_{x,x^{\prime \prime }})\; & \text{if}\;{\text{deg}}_{\mathrm{out}}x^{\prime }>1\;\text{and}\\ \delta_{x,x^{\prime \prime }} & \text{if}\;{\text{deg}}_{\mathrm{out}}x^{\prime }=1\;\text{and} \end{array} & x^{\prime }\to x\;\text{is possible}\\ \frac{A_{x^{\prime }x^{\prime \prime }}}{\sum_{\xi }A_{x^{\prime }\xi }} & \text{otherwise}, \end{array}\right. \end{array}$$

where the persistence q∈[0,1] controls the probability of returning to the previous site in the sense that 1−q is the probability of turning around. With the transition kernel for a standard random walk,


(5) $$\kappa_{0}(x^{\prime \prime }|x^{\prime },x)=\frac{A_{x^{\prime }x^{\prime \prime }}}{\sum_{\xi }A_{x^{\prime }\xi }}$$

we define the transition kernel


(6) $$\kappa (x^{\prime \prime }|x^{\prime },x)=(1-\nu )\kappa_{0}(x^{\prime \prime }|x^{\prime },x)+\nu \kappa_{\mathrm{inertial}}(x^{\prime \prime }|x^{\prime },x)$$

where the parameter ν∈[0,1] allows us to interpolate between the random walk with (ν=1) and without inertia (ν=0).

Suppose $({\hat{X}}_{n}{)}_{n}$ is a discrete-time random walk on this Markov transition network and let *Π* be the projection from the Markov transition network to a node or link in the underlying transport network, i.e. Π(Ω)=Ω, $\Pi (x_{\mathrm{in}})=x=\Pi (x_{\mathrm{out}})$, and $\Pi ((x,x^{\prime }{)}^{\left(l\right)})=(x,x^{\prime })$ for every *l*, then a walker’s trajectory under this mobility model is given by a process $(X_{n}{)}_{n}$ with $X_{n}\;:\;=\Pi ({\hat{X}}_{n})$ and ${\hat{X}}_{0}=\Omega$. Since $s_{l}\to 1$ as l→∞ any trajectory will eventually reach *Ω* again, producing an sequence of the form $\Omega ,x,(x,x^{\prime }),(x^{\prime },x^{\prime \prime }),\ldots (x^{\prime \cdots \prime },x^{\prime \cdots \prime \prime }),x^{\prime \cdots \prime \prime },\Omega$. An *excursion* under this mobility model is the corresponding sequence of traversed sites, $x,x^{\prime },\ldots x^{\prime \cdots \prime },x^{\prime \cdots \prime \prime }$ we will say that it has *l* steps if it consists of l+1 sites.

By construction, the transitions along the excursions appear as if they were drawn from the transition kernel $\kappa (x^{\prime \prime }|x^{\prime },x)$ (6) and the probability that any excursion has length *l* is $\sigma_{l}$ (as long as there are no nodes *x* with ${\text{deg}}_{\mathrm{out}}x=0$ in the transport network, otherwise consider only the excursions that do not end at one of those sites).

For this work, we used the Munich bus network with its 986 nodes and 2,228 arcs that has been constructed as described above. After multiple arcs between the same nodes have been collapsed into a single accordingly weighted arc this became the base network. Furthermore, we set parameter values q=0.95, ν=1, and $\sigma_{l}\propto \exp\;(-(\frac{l-8.85907}{16.0027}{)}^{2})$ for 1≤l≤49 such that $\sum_{l=1}^{49}\sigma_{l}=1$. The latter distribution has been found empirically. Moreover, the entry distribution *α* was derived from the MCNF access distribution and thus linked to the overall travel demand. Finally, to estimate the travel time, we assumed an average speed of 25 km/h. Given this model, we simulated $10^{7}$ excursions.

## Data Availability

The data that support the findings of this study is available through figshare Doi: 10.6084/m9.figshare.27284223.
